# Cures for the Cholera

**Published:** 1848-10

**Authors:** 


Cures for the Cholera.—One good must accrue from the newspaper discussions of this impending calamity, and that is, the salutary fear which they must create of any attempt to treat the disease without competent medical superintendence. Already we suspect the prudence, of adopting the every-man-his-own-doctor plan, begins to be doubtful, and cholera-curing amateurs begin to waver. The men of "little faith in physics," too, are looking uneasy, and the advocates of cheap doctoring somewhat sheepish. But what's to come of the homœopathists? Are they lying in a store of globules, and preparing to display to the death their conviction that the millionth part of a grain of opium is more powerful than the entire grain? Then the hydropaths are, we conclude, preparing a full supply of sheets for cutaneous refrigeration, and a plentiful one of water for gastric dilution. Altogether the times are favorable for testing faiths and fancies.—*Dublin Medical Press.*

*Memorandum of the Arabian Prescription of Asafœdita, Opium, &c. in Cholera.*

This ancient remedy for the most destructive disease of either ancient or modern times does not consist of rare or numerous ingredients. They are articles in common use by all Asiatics, and found in every bazaar (market) in Asia—namely, asafœtida, opium, black pepper (pulverised.) I continued the use of this medicine for years, during my residence in India, with the greatest success, the extent of which will be best understood when I say that, instead of despair, as formerly when a case of cholera was brought to me, I learned to feel confident that if the patient was not so far gone that the vital powers were well nigh extinct, his life might be saved.

*Extract of a Letter from Mr. Thomas Wise, M. D., dated Dakkah, April 2, 1847.*

Many years ago you asked me my opinion of your prescription (the asafœdita and opium pills in cholera.) My report was then favorable, and I think you will be pleased to know the result of my further experience. I am happy to say it is still very favorable: indeed, so much so, that when they (the pills) are given in an early stage of the disease, they almost always accomplish a cure. So much is this the case that I always use them; and since September last, during the prevalence of a prevalent epidemic, I find I have distributed 20,600 pills. Almost every turning in the city of Dakkah had boards indicating where the pills were to be got. When the patients were brought to the hospital, or when I see them first in a collapsed state of cholera, I give a pill broken down (bruised in a spoonful of brandy and water,) and repeat it; and I apply the tourniquet to the four extremities. This throws several pounds of blood into the trunk, and disturbs and removes the morbid action; and thus patients are cured when there is no chance with any other remedies.

The tourniquet is a recent application, to which Dr. Wise attaches considarable importance.

### *Mode of Using the Medicine.*

Ingredients for one dose for an adult:—Asafœtida, opium, black pepper (pulverised,) of each two grains made into a pill.

N. B.—Should two grains of opium be thought too large a dose (which if very pure opium be used it may be,) 1½ grains may be tried.

These pills may be made up and kept for use in a phial, the mouth of it being well closed. When used the pills should be broken down and bruised and taken in a table-spoonful of brandy and water, and washed down with a small quantity of the same. (It would be still better to chew the pill and swallow it, washed down in the same manner.) But the pills should not be swallowed whole, as they would not act so promptly, and might be brought up by vomiting. The dose should be repeated every half-hour* or hour, according to the urgency of the case, until the symptoms be subdued. Two or three doses are generally sufficient, but five or more have been given before the disease has been arrested, giving half or quarter doses at shorter intervals; and in cases of great prostration and protracted disease I have, as an additional stimulant, substituted red pepper for the black pepper occasionally.

Friction, with hot and stimulating substances, over the stomach and abdomen, should also be used. The limbs also should be well rubbed in the same way; and if the patient has complained of more than usual pain in the stomach I have sometimes given ten grains of calomel—although I cannot say that I have observed much benefit from it, unless where there has been congestion of liver; or indeed from anything taken internally except this medicine. If there be much thirst, as generally there is, a few spoonsful of brandy and water may be given.

In cases of collapse the same course must be pursued and continued, the medicine being repeated at intervals of longer or shorter duration, according to the state of the patient. And as Dr. Wise has recommended the application of the tourniquet to the arms and legs in order to husband as it were the vital power by limiting the extent of circulation, this may be tried by applying a ligature of tape or other substances to the upper arm and thigh if the tourniquet be not available.—*Medical Gazette.—Dublin Medical Press.*

---

*Progress of the Cholera.*—The spasmodic or Asiatic Cholera, continues to be the great theme of discussion in our European exchange journals, and as it may, like former epidemics, extend to this continent, the subject, in its various bearings, is one of much interest to us also. That it will make its appearance again in America, at no very distant period, is ren-

* Two grains of opium every half hour, to the extent of five doses, would be heroic practice. The patient may recover from the disease, but die from the treatment.—*Ed. Gaz.*

